# Adaptive mechanisms of *Campylobacter jejuni* to erythromycin treatment

**DOI:** 10.1186/1471-2180-13-133

**Published:** 2013-06-14

**Authors:** Qingqing Xia, Wayne T Muraoka, Zhangqi Shen, Orhan Sahin, Hongning Wang, Zuowei Wu, Peng Liu, Qijing Zhang

**Affiliations:** 1 Animal Disease Prevention and Food Safety Key Laboratory of Sichuan Province, “985”Project Science Innovative Platform for Resource and Environment Protection of Southwestern, Key Laboratory of Bio-resources and Eco-environment of Ministry of Education, School of Life Science, Sichuan University, Chengdu, Sichuan, 610064, P. R. China; 2 Department of Veterinary Microbiology and Preventive Medicine, Iowa State University, Ames, IA, 50011, USA; 3 Department of Statistics, Iowa State University, Ames, IA, 50011, USA

**Keywords:** Adaptation, Microarray, Transcriptome, Macrolide, *Campylobacter*

## Abstract

**Background:**

Macrolide is the drug of choice to treat human campylobacteriosis, but *Campylobacter* resistance to this antibiotic is rising. The mechanisms employed by *Campylobacter jejuni* to adapt to erythromycin treatment remain unknown and are examined in this study. The transcriptomic response of *C. jejuni* NCTC 11168 to erythromycin (Ery) treatment was determined by competitive microarray hybridizations. Representative genes identified to be differentially expressed were further characterized by constructing mutants and assessing their involvement in antimicrobial susceptibility, oxidative stress tolerance, and chicken colonization.

**Results:**

Following the treatment with an inhibitory dose of Ery, 139 genes were up-regulated and 119 were down-regulated. Many genes associated with flagellar biosynthesis and motility was up-regulated, while many genes involved in tricarboxylic acid cycle, electron transport, and ribonucleotide biosynthesis were down-regulated. Exposure to a sub-inhibitory dose of Ery resulted in differential expression of much fewer genes. Interestingly, two putative drug efflux operons (*cj0309c-cj0310c* and *cj1173-cj1174*) were up-regulated. Although mutation of the two operons did not alter the susceptibility of *C. jejuni* to antimicrobials, it reduced *Campylobacter* growth under high-level oxygen. Another notable finding is the consistent up-regulation of *cj1169c-cj1170c,* of which *cj1170c* encodes a known phosphokinase, an important regulatory protein in *C. jejuni*. Mutation of the *cj1169c-cj1170c* rendered *C. jejuni* less tolerant to atmospheric oxygen and reduced *Campylobacter* colonization and transmission in chickens.

**Conclusions:**

These findings indicate that Ery treatment elicits a range of changes in *C. jejuni* transcriptome and affects the expression of genes important for *in vitro* and *in vivo* adaptation. Up-regulation of motility and down-regulation of energy metabolism likely facilitate *Campylobacter* to survive during Ery treatment. These findings provide new insight into *Campylobacter* adaptive response to antibiotic treatment and may help to understand the mechanisms underlying antibiotic resistance development.

## Background

*Campylobacter jejuni* is a Gram-negative, spiral-shaped, motile bacterium and is a leading cause of bacterial food-borne enteritis in humans [1,2]. Most human *C. jejuni* infections are acquired by consuming or handling contaminated poultry, milk or water. Clinical symptoms of campylobacteriosis can range from mild diarrhea to fever, headache, abdominal cramping, vomiting and bloody diarrhea. Studies also demonstrated that *Campylobacter* infection is associated with Guillain-Barré syndrome as a post-infection complication [3].

Although most campylobacteriosis cases are self-limiting, antibiotic therapy may be necessary for severe or persistent illness [4]. Macrolide, such as erythromycin (Ery), is the drug of choice for treating campylobacteriosis, but the frequency of resistance to this class of antibiotic is rising [5,6]. As an inhibitor of protein translation in bacterial cells, Ery and other macrolide antibiotics interfere with aminoacyl translocation, preventing the transfer of the tRNA bound at the A site to the P site of the rRNA complex. Without this translocation, the A site remains occupied and thus precludes the incoming tRNA from attaching its amino acid to the nascent polypeptide [7-9]. The molecular mechanism of resistance to Ery in *C. jejuni* has been extensively studied and is conferred largely by target modification (such as mutations in the 23S rRNA gene and ribosomal proteins) [6,7,10] and antibiotic efflux pumps [11]. Although the genetic basis of Ery resistance in *C. jejuni* has been well characterized, there is very little knowledge of the initial response and adaptive mechanism of *C. jejuni* to Ery exposure.

Transcriptomic analysis has been used to assess bacterial adaptive responses to antibiotic treatments. Three previous studies reported global gene expression patterns of *Streptococcus pneumonia*[12], *Escherichia coli*[13], and *Haemophilus influenzae*[14] to sub-inhibitory doses of translation-inhibiting antibiotics. These reports demonstrated that exposure to these bacteriostatic antibiotics triggered the synthesis of a number of ribosomal proteins [12-14]. Other studies analyzed the transcriptional profiles of *Staphlococcus aureus*, *E. coli*, and *Yersinia pestis* under inhibitory doses of chloramphenicol, mupirocin, ampicillin, or ofloxacin [15-17], and a common observation of these studies was the repression of energy metabolism genes by these antibiotics. Although the transcriptomic response of *C. jejuni* to a fluoroquinolone antibiotic has been reported [18], it remains unknown how this organism responds to macrolide treatment.

In this study, the genome-wide transcriptional response of *C. jejuni* following exposure to both inhibitory and sub-inhibitory doses of Ery was assessed. Furthermore, contribution of several differentially expressed genes to antibiotic resistance, stress resistance, and host colonization was determined using isogenic gene knock-out mutants.

## Results

### Transcriptional responses of NCTC 11168 to an inhibitory dose of Ery

To identify the adaptive response of *Campylobacter* to Ery treatment, microarray was used to analyze the transcriptional changes in *C. jejuni* NCTC 11168 following exposure to Ery. After NCTC 11168 was exposed to an inhibitory dose of Ery (16× MIC) for 30 min, a total of 258 genes were shown to be differentially expressed, among which 139 were up-regulated and 119 were down-regulated (Additional file 1: Tables S1 and S2). Cluster of orthologous groups (COG) (http://www.ncbi.nlm.nih.gov/COG/) analysis revealed changes in multiple functional categories (Table 1). Among the up-regulated genes, the “cell motility” category showed the highest percentage (19.23%) of changes. For the down-regulated genes, the “Energy production and conversion” category showed the highest percentage (31.58%) of changes. Additionally, a number (85; 33%) of the differentially expressed genes were in the categories of “poorly characterized”/“function unknown”/”General function prediction only” (Table 1).

**Table 1:** COG category of differentially-expressed genes in NCTC 11168 in response to treatment with an inhibitory dose of Ery

**COG category**	No. up-regulated (%)*	No. down-regulated (%)*	**Total No. differentially expressed genes**
Amino acid transport and metabolism	14 (11.11%)	12 (9.52%)	26
Carbohydrate transport and metabolism	1 (2.94%)	4 (11.76%)	5
Cell cycle control, mitosis and meiosis	2 (14.29%)	2 (14.29%)	4
Cell motility	10 (19.23%)	2 (3.85%)	12
Cell wall/membrane biogenesis	3 (2.52%)	9 (7.56%)	12
Coenzyme transport and metabolism	7 (10.14%)	3 (4.35%)	10
Defense mechanisms	2 (8.70%)	0 (0.00%)	2
Energy production and conversion	6 (6.32%)	30 (31.58%)	36
Function unknown	9 (12.67%)	3 (4.23%)	12
General function prediction only	12 (8.45%)	10 (7.04%)	22
Intracellular trafficking and secretion	0 (0.00%)	1 (2.17%)	1
Inorganic ion transport and metabolism	9 (11.11%)	4 (4.94%)	13
Lipid transport and metabolism	3 (8.57%)	0 (0.00%)	3
Nucleotide transport and metabolism	1 (2.33%)	4 (9.30%)	5
Poorly characterized	32 (6.00%)	19 (3.56%)	51
Posttranslational modification, chaperones	6 (9.23%)	7 (10.77%)	13
Replication, recombination and repair	3 (5.00%)	3 (5.00%)	6
Signal transduction mechanisms	3 (6.67%)	1 (2.22%)	4
Transcription	6 (13.95%)	1 (2.33%)	7
Translation	10 (10.00%)	4 (4.00%)	14
Total	139	119	258

* This percentage was calculated based on the number of the up or down regulated genes in a category to the total number of the genes in that particular category.

Within the up-regulated genes, several belong to putative transcriptional units (operons) including *cj0061c-cj0062c*, *cj0309c-cj0310c*, *cj0345-cj0349*, *cj0423-cj0425*, *cj0951c-cj0952c*, and *cj1173-cj1174*. *cj0061c* encodes a flagellar biosynthesis sigma factor and *cj0062c* encodes a putative integral membrane protein. Each of the *cj0309c-cj0310c* and *cj1173-cj1174* operons encodes a putative multidrug efflux system in *C. jejuni*. Genes *cj0345-cj0349* are predicted to encode subunits of anthranilate synthase and tryptophan synthase. *cj0423-cj0425* encode putative integral membrane/periplasmic proteins whose functions remain unknown. *cj0951c-cj0952c* encode proteins forming a putative chemoreceptor, which was demonstrated to be associated with host cell invasion, motility and chemotaxis towards formic acid [19].

Many of the down-regulated genes belonged to the “energy production and conversion” category (Table 1). Approximately 31.58% (30 out of 95) of the genes classified in “energy production and conversion” were down-regulated in response to the inhibitory Ery treatment. Included in this category were several putative operons, such as *cj0073c-cj0076c*, *cj0107-cj0108*, *cj0437-cj0439*, *cj0531-cj0533*, *cj0781-cj0783*, *cj1184c-cj1185c*, *cj1265c-cj1266c*, and *cj1566-cj1567*. Several ORFs in other COGs also showed a substantial level of down-regulation and these included *cj0662c-cj0663c*, which encode an ATP-dependent protease ATP-binding subunit HslU and an ATP-dependent protease peptidase subunit; *cj1427c-cj1428c,* which encode two proteins belonging to carbohydrate transport and metabolism; and *cj1598-cj1599,* which encode two amino acid transport and metabolism proteins.

### Transcriptional responses of NCTC 11168 to a sub-inhibitory dose of Ery

To identify differentially expressed genes in response to a sub-inhibitory concentration of Ery, microarray was performed on wild-type *C. jejuni* NCTC 11168. In total, the expression of 85 genes was altered by the sub-inhibitory dose (0.5 × MIC) of Ery treatment, of which 39 were up-regulated and 46 were down-regulated (Table 2; Additional file 1: Tables S3 and S4). More than half (50.59%) of the differentially expressed genes encoded hypothetical proteins (included “poorly characterized”/“function unknown”/”General function prediction only”). Several differentially expressed genes were in the functional category of “amino acid transport and metabolism” (6 were up-regulated and 5 were down-regulated) (Table 2). The up-regulated genes in this category included *trpB*, *trpD*, *trpA*, *trpE* (*cj0348*, *cj0346*, *cj0349*, *cj0345*) encoding tryptophan synthase and anthranilate synthase subunits, two genes (*cj1017c*, *cj1019c*) encoding a branched-chain amino-acid ABC transport system permease and a periplasmic binding proteins. Down-regulated genes in this category included *argB* (*cj0226*), *cysE* (*cj0763c*), *cj0731*, *cj1582c,* and *cj1583c*. Fewer than 3 genes were differentially expressed in other categories (Table 2). Different from the inhibitory treatment, the sub-inhibitory treatment resulted in much fewer differentially expressed genes in the “transcription” and “translation” categories (Table 2).

**Table 2:** COG category of differentially-expressed genes in NCTC 11168 in response to treatment with a sub-inhibitory dose of Ery

**COG category**	No. up-regulated (%)*	No. down-regulated (%)*	**Total No. differentially expressed genes**
Amino acid transport and metabolism	6 (4.76%)	5 (3.97%)	11
Carbohydrate transport and metabolism	1 (2.94%)	2 (5.88%)	3
Cell motility	2 (3.85%)	0 (0.00%)	2
Cell wall/membrane biogenesis	0 (0.00%)	3 (2.52%)	3
Coenzyme transport and metabolism	1 (1.45%)	2 (2.90%)	3
Defense mechanisms	1 (4.35%)	1 (4.35%)	2
Function unknown	4 (5.63%)	3 (4.23%)	7
General function prediction only	2 (1.41%)	2 (1.41%)	4
Inorganic ion transport and metabolism	3 (3.70%)	2 (4.94%)	5
Lipid transport and metabolism	1 (2.86%)	2 (5.71%)	3
Poorly characterized	15 (2.81%)	17 (5.71%)	32
Posttranslational modification, chaperones	0 (0.00%)	1 (1.54%)	1
Replication, recombination and repair	0 (0.00%)	1 (1.67%)	1
Signal transduction mechanisms	1 (2.22%)	2 (4.44%)	3
Transcription	2 (4.65%)	2 (4.65%)	4
Translation	0 (0.00%)	1 (1.00%)	1
Total	39	46	85

* This percentage was calculated based on the number of the up or down regulated genes in a category to the total number of the genes in that particular category.

Notably, several genes demonstrated consistent changes in expression under both inhibitory and sub-inhibitory treatments with Ery and are listed in Table 3. These genes are involved in motility/chemotaxis, tryptophan synthesis, branched-chain amino acid transport, and protein phosphorylation (*cj1170c)*. A two-component sensor kinase (*cj1226c*) was down-regulated under both inhibitory and sub-inhibitory treatments (Table 3). To confirm differential expression detected by microarray, qRT-PCR was conducted on selected genes. The result confirmed most of the examined genes (Table 4).

**Table 3:** Differentially-expressed genes in NCTC 11168 in response to inhibitory/sub-inhibitory Ery treatments

**Gene name**	**Description**	**Fold-change**	
		**Inhibitory treatment**	**Sub-inhibitory treatment**
*cj0061c*	flagellar biosynthesis sigma factor	4.44	3.03
*cj0345*	putative anthranilate synthase component I	7.84	5.02
*cj0348*	tryptophan synthase subunit beta	4.51	2.76
*cj0565*	Pseudogene	6.12	4.17
*cj0698*	flagellar basal body rod protein FlgG	5.10	3.45
*cj0916c*	conserved hypothetical protein Cj0916c	4.43	3.29
*cj0951c*	putative MCP-domain signal transduction protein	5.75	4.44
*cj0952c*	putative HAMP containing membrane protein	7.85	2.84
*cj1019c*	branched-chain amino-acid ABC transport system periplasmic binding protein	12.11	3.13
*cj1169c*	putative periplasmic protein	6.91	2.71
*cj1170c*	50-KDa outer membrane protein precursor	15.34	2.75
*cj0168c*	putative periplasmic protein	0.08	0.29
*cj0767c*	phosphopantetheine adenylyltransferase	0.23	0.24
*cj1226c*	putative two-component sensor (histidine kinase)	0.29	0.30

**Table 4:** qRT-PCR confirmation of representative differentially expressed genes initially identified by microarray

**Gene**	**Ery**-**treatment**	**qRT-PCR**		**Microarray**	
		FC**	P* value	**FC**	P* value
*cj0061c*	Inhibitory	7.92	0.01	4.44	0.01
*cj0061c*	Sub-inhibitory	2.60	0.03	3.03	0.01
*cj0258*	Inhibitory	0.71	0.35	0.70	0.43
*cj0258*	Sub-inhibitory	2.33	0.08	6.88	0.01
*cj0310c*	Inhibitory	2.77	0.05	5.49	0.01
*cj0310c*	Sub-inhibitory	2.07	0.02	1.82	0.14
*cj0345*	Inhibitory	29.10	0.01	7.84	0.01
*cj0345*	Sub-inhibitory	6.94	0.03	3.93	0.01
*cj0425*	Inhibitory	6.80	0.01	107.44	0.01
*cj0425*	Sub-inhibitory	6.61	0.01	2.01	0.05
*cj1170*	Inhibitory	55.71	0.01	15.34	0.01
*cj1170*	Sub-inhibitory	4.21	0.17	2.75	0.01
*cj1173*	Inhibitory	6.38	0.02	4.31	0.01
*cj1173*	Sub-inhibitory	3.65	0.01	1.43	0.19
*cj1226*	Inhibitory	0.07	0.01	0.29	0.01
*cj1226*	Sub-inhibitory	1.72	0.29	0.31	0.01
*cj1563*	Inhibitory	1.95	0.03	4.97	0.01
*cj1563*	Sub-inhibitory	1.61	0.01	0.86	0.53

* P values smaller than 0.01 are shown as 0.01.

** FC denotes fold-change.

### Transcriptional responses of Ery^R^*C. jejuni* JL272 to Ery treatment

JL272 is an Ery^R^ derivative of NCTC 11168 and was isolated from a chicken fed tylosin-containing feed [20]. This strain bears a A2074G mutation in its 23S rRNA gene, which confers a high-level erythromycin resistance (MIC = 1024 mg/L) [20]. The transcriptional profile of this strain was assessed after treatment with 4 mg/L of Ery, the same concentration used for the inhibitory treatment of the wild-type strain. Interestingly, only a total of three genes were up-regulated, while a single gene was down-regulated. The up-regulated genes were *cj0862c*, *cj1006c* and *cj1706c*, which encode para-aminobenzoate synthase component I, a hypothetical protein and 50S ribosomal subunit protein RplD, respectively. The down-regulated gene, *cj0030*, encodes a hypothetical protein. The small number of affected genes in the Ery^R^ strain suggests that little stress is imposed to JL272 by 4 mg/L of Ery.

### Characterization of *cj0309c-cj0310c* and *cj1173-cj1174*

Two of the operons up-regulated by Ery treatment were *cj0309c*-*cj0310c* and *cj1173-cj1174,* which encode putative small multidrug resistance (SMR) efflux transporters. However, their functions have not been determined. The SMR family of transporters are characterized by their short length (100–150 amino acids), four trans-membrane α-helical motifs, and the use of the proton motive force to export a broad range of antiseptics and drugs out of the cell [21]. The paired small multidrug resistance (PSMR) protein family is one of the SMR sub-classes, which requires co-expression of two homologues including a typical SMR length protein and a protein with longer hydrophilic loops [22].

**Figure 1:**
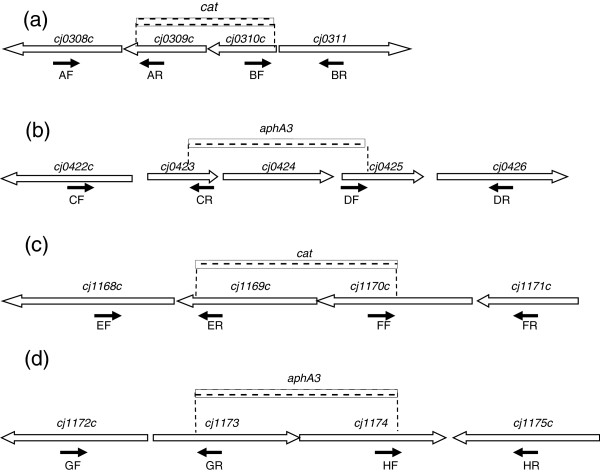
**Construction of mutant strains.** ORFs are indicated by boxed arrows (not drawn to scale). The locations of the primers used to amplify the fragments and generate the deletions are indicated by solid arrows. The dash line box indicated the location of the deletion of chromosomal sequence and insertion of an antibiotic resistant cassette (*cat* or *aphA3*). (**a**), (**b**), (**c**), and (**d**) are diagrams for operons *cj0309c-cj0310c*, *cj0423-cj0425*, *cj1169c-cj1170c* and *cj1173-cj1174*, respectively.

*Cj0309c-cj0310c* and *cj1173-cj1174* belong to the PSMR family. In the microarray experiment, both pairs were up-regulated in response to the inhibitory dose of Ery treatment (Additional file 1: Table S1 and Table 4). To determine the role of the PSMR genes in adaptive response to Ery exposure, *C. jejuni* NCTC 11168 mutants carrying a mutation(s) in either (single mutant) or both (double mutant) PSMR operons were constructed (Table 5, Figure 1). None of the mutant strains had any substantial *in vitro* growth defect compared to the wild-type strain in MH broth after 48 hours of incubation under microaerobic conditions (data not shown). Mutation of the PSMR transporter genes, either individually or in combination, did not substantially change the MIC toward 14 compounds tested, including Ery (data not shown; see materials and methods for the compounds tested).

**Table 5:** Bacterial strains and plasmids used in this study

**Strain or plasmid**	**Description or relevant genotype**	**Source or reference**
Bacterial strains		
NCTC 11168	*Campylobacter jejuni* wild-type strain	ATCC*
JL272	NCTC 11168 derivative erythromycin resistant strain	Jun Lin [20]
KOp50Q	NCTC 11168 Δ*cj1169c-cj1170*c::*cat* mutant	This study
KO423Q	NCTC 11168 Δ*cj0423-cj0425*::*aphA3* mutant	This study
KO39Q	NCTC 11168 Δ*cj0309c-cj0310*c::*cat* mutant	This study
KO73Q	NCTC 11168 Δ*cj1173-cj1174*::*aphA3* mutant	This study
DKO01Q	NCTC 11168 Δ*cj0309c-cj0310*::*cat*, Δ*cj1173-cj1174*::*aphA3* mutant	This study
Comp50Q	KOp50Q complementation strain; Δ*cj1169c-cj1170*c::*cat*, ITS1::*cj1169c-cj1170c* complementation strain	This study
ComDK01Q	DKO01Q complementation strain; Δ*cj0309c-cj0310*::*cat*, Δ*cj1173-cj1174*::*aphA3*, ITS1*::cj1173-cj1174*	This study
DH5α	*Escherichia coli* strain for plasmid propagation	Invitrogen
Plasmids		
pUC19	Polymerase chain reaction cloning vector, Amp^R**^	Promega
pRR	Complementation cloning vector	Karlyshev [40]
pRRK	pRR with *aphA3*; Kan^R**^	This study
pRRT	pRR with *tetO*; Tet^R**^	This study
pUp50Q	*cj1169c-cj1170*c::*cat* cloned into pUC19	This study
pU423Q	*cj0423-cj0425*::*aphA3* cloned into pUC19	This study
pU39Q	*cj0309c-cj0310c*::*cat* cloned into pUC19	This study
pU74Q	*cj1173-cj1174*::*aphA3* cloned into pUC19	This study
pRRKp50Q	*cj1169c-cj1170*c cloned into pRRK	This study
pRRK39Q	*cj0309c-cj0310c* cloned into pRRK	This study
pRRT73Q	*cj1173-cj1174* cloned into pRRT	This study

*ATCC: American type culture collection (ATCC number for this strain is 700819).

**Amp^R^: Ampicillin resistance, Kan^R^: Kanamycin resistance, Tet^R^: Tetracycline resistance.

**Figure 2:**
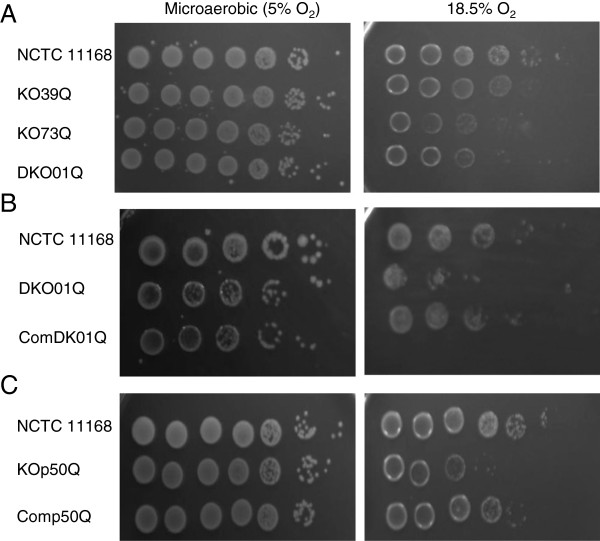
**Comparison of oxygen tolerance of *C. jejuni *wild-type NCTC 11168 and its mutant strains.** For (**A**) and (**C**), 5 μl of serial dilutions (from left to right: 10^7^-10^1^ CFU/ml) of overnight cultures were spotted onto MH agar plates and incubated at either 18.5% or 5% O_2_. For (**B**), 5 μl of serial dilutions (from left to right: 10^5^-10^1^ CFU/ml) of overnight cultures were spotted onto MH agar plates and incubated at either 18.5% or 5% O_2_. Results are representative of three independent experiments.

The involvement of the PSMR efflux systems in aerobic and oxidative stress survival in *C. jejuni* was tested next. In this experiment, the ability of bacterial cells to grow on MH agar was assessed under different oxygen levels (5% O_2_ or 18.5% O_2_). The PSMR mutants and their wild-type strain grew comparably under microaerobic environment (5% O_2_) (Figure 2A). However, under aerobic conditions (18.5% O_2_), all mutants showed declined growth compared with the wild-type strain (Figure 2A) and the decline was more prominent with KO73Q and DKO01Q (~100 fold difference). To confirm the phenotype associated with the mutant strains, a partial complementation of the double knock-out mutant with the wild-type copy of *cj1173-cj1174* was constructed as described in material and methods. As shown in Figure 2B, the complementation partly restored the mutant’s ability to grow under high oxygen tension. These results indicated that the two PMSR systems facilitate *C. jejuni* adaptation to aerobic environment. Additionally, we performed disk diffusion assay using hydrogen peroxide, cumene, and menadione, which did not show any significant differences (*p* > 0.05) in bacterial growth inhibition between the wild-type and PSMR mutant strains (result not shown), suggesting that the two putative efflux systems are not directly involved in the resistance to the examined oxidants.

**Figure 3:**
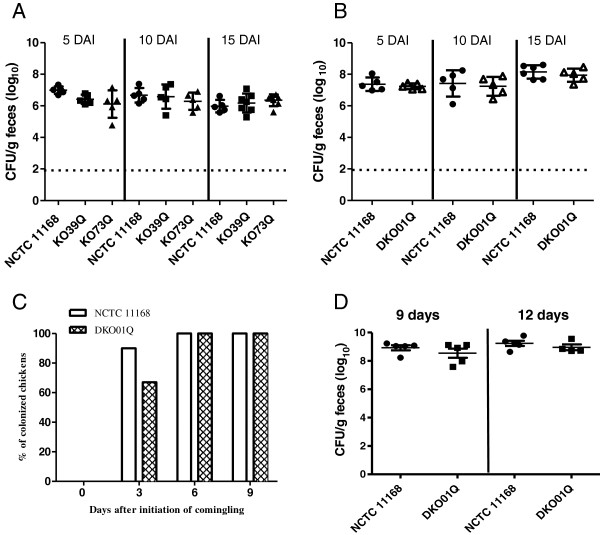
**Effect of the PSMR gene mutations on *Campylobacter *colonization and transmission in chickens.** (**A**) Colonization levels of single-mutant strains KO39Q and KO73Q in comparison with the wild-type strain NCTC11168. (**B**) Colonization levels of double mutant DKO01Q in comparison with the wild-type strain NCTC11168. In (**A**) and (**B**), cecal contents were collected from chickens necropsied on DAI 5, 10, and 15. Each symbol represents data from a single bird and bars indicate the mean ± SD for each group. Dashed lines indicate the detection limit of the direct plating method. (**C**) and (**D**): Co-mingling experiment demonstrating the transmission of *C. jejuni* from seeder birds (n = 3 in each group) to naive (non-inoculated) birds. (**C**) The percentage of naive birds (n = 10 for the wild type group and n = 9 for DKO01Q group) positive for *C. jejuni* after comingling with seeder birds inoculated with NCTC11168 and DKO01Q, respectively. (**D**) Cecal colonization levels of the wild-type strain and DKO01Q strains in naive birds co-mingled with the seeder birds. The birds were euthanized at 9 and 12 days after initiation of co-mingling. Each symbol represents the colonization level of a single bird and the horizontal bars indicate the mean and standard error for each group.

Since the PSMR mutants demonstrated enhanced susceptibility to the high-level oxygen concentration, we further examined their contribution to colonization of chickens. Both the wild-type and the mutant strains were equally motile as determined by swarming on semi-solid agar. When chickens were mono-inoculated individually with each mutant strain (KO39Q, KO73Q or DKO01Q), there was no significant difference in the level of colonization among the wild-type and mutant strains for the duration of study, i.e., 15 days after inoculation (Figure 3A and B). Additionally, a co-mingling chicken experiment using the double knockout mutant and wild-type strain was performed in order to determine the role of the PSMR genes in horizontal transmission in birds. In the comingling group with seeder birds inoculated with the double knockout mutant, 67% of the naive chickens were positive for DKO01Q at 3 days after initiation of co-mingling, and all the birds became positive at 6 and 9 days after initiation of co-mingling (Figure 3C). For the comingling group with seeder birds inoculated with the wild-type strain, 90% of the naive birds were colonized with NCTC 11168 at 3 days after initiation of comingling, and all colonized at 6 and 9 days after initiation of comingling (Figure 3C). The colonization levels in the non-inoculated, but comingled birds also showed no significant differences between the two groups (Figure 3D). Together, the chicken experiments indicated that the two PSMR efflux systems, individually or in combination, are dispensable for *C. jejuni* colonization and horizontal spread in the chicken host.

### Characterization of the *cj0423-cj0425* operon

*cj0423-cj0425* encode a putative integral membrane protein, a putative acidic periplasmic protein and a putative periplasmic protein, respectively. Microarray showed that this operon was up-regulated under treatment with an inhibitory dose of Ery (Additional file 1: Table S1). Additionally, qRT-PCR results demonstrated that *cj0425* was up-regulated under both inhibitory and sub-inhibitory Ery treatments in NCTC 11168 (Table 4). Amplification of *cj0423-cj0425* by a conventional RT-PCR (primers are listed in Table 6) confirmed that *cj0423*-*cj0425* were co-transcribed (data not shown), suggesting an operon-like structure. To characterize the function of this operon, all three genes were deleted to generate mutant KO423Q as described in materials and methods. The mutation did not affect the transcript abundance of the downstream gene (*cj0426*) as qRT-PCR revealed no significant difference in the transcript quantity of *cj0426* between the wild-type and the mutant strain (*p* = 0.07). When the wild-type strain and KO423Q were compared for *in vitro* growth in MH broth, there were no significant growth rate differences at 24 h and 48 h (data not shown). In addition, Ery MIC of KO423Q was the same as that of the wild-type strain (0.25 mg/L). Moreover, no appreciable difference was evident for oxidative stress resistance (H_2_O_2_, cumene hydroperoxide, and menadione) between the wild-type and the mutant strains (results not shown).

**Table 6:** Primers used in this study

**Primers**	Sequence*(5’ → 3’) (restriction enzyme)
Primers for mutant construction	
cj0308c-F(AF)	CTCGAATTCCCTTTTCCATAGATGTTTGC (EcoRI)
cj0309c-R (AR)	CCAGGTACCAAATTTCACAGGCAGTAGCT(KpnI)
cj0310c-F (BF)	GAATCTAGAGTGGGGTAATGATAGGAGTG(XbaI)
cj0311-R (BR)	CCAGTCGACCAAGGGCTAAAAGATTGATA (SalI)
cj0422c-F (CF)	GCCGAATTCAATAAGGGTTGTAATTGAAGCG (EcoRI)
cj0423-R (CR)	AATGGTACCTGCTCAAACCAATAATAGCG(KpnI)
cj0425-F (DF)	TTTGCTAGCGGACTA TTATTGTTTGGCTTGT (NheI)
cj0426-R (DR)	ATCCTGCAGGTCTAATGAGTTGGCCTGAA (PstI)
cj1168c-F(EF)	AGCGAATTCACAGAACCTAAAGTCCCA (EcoRI)
cj1169c-R (ER)	AATCGGTACCATCCAGAGCCAGAGTCAAA(KpnI)
cj1170c-F (FF)	AAATCTAGAATGAAAGAAGACAAGCACT(XbaI)
cj1171c-R (FR)	TTGGCATGCTAGAAACTGAAAAAGGCAC (SphI)
cj1172c-F (GF)	ACAGAATTCACGGTGCGTGTAGGGTT (EcoRI)
cj1173-R (GR)	ACTGGTACCACAATAGCAGCAATCAAGAA(KpnI)
cj1174-F (HF)	GGTTCTAGAGGCATCATAGGAACTTGTCT(XbaI)
cj1175c-R (HR)	GGTCTGCAGATCGATGATGTAATGAAAGC (PstI)
16S-F	ATCGTAGATCAGCCATGCTA
Primers for complementation construction	
*cj1169c-cj1170c*-F	TTGTCTAGAAAAAGATTAAACAGTAA(XbaI)
*cj1169c-cj1170c*-R	AACTCTAGAACAAGGAGTTATGATTA(XbaI)
*cj1173-cj1174*-F	TATTCTAGACAGTGCCACCTTCTTTAGCG(XbaI)
*cj1173-cj1174*-R	CCGTCTAGACTTTAATGGGTATTGAAGCA(XbaI)
Primers for qRT-PCR	
qRT-16S-F	TCCCAGTTCGGATTGTTCTC
qRT-16S-R	GTACAAGACCCGGGAACGTA
cj0061-F	GAAGGCAAGCGTTCTTTTAGTCT
cj0061-R	AGAAAGAGCAAGATGAGCTTGTG
cj0258-F	GTGAAAATGCCGATGAAAATG
cj0258-R	GCGATGATGGTAGGTTTACTTTG
cj0310c-F	TAGAGTGCTTTTGGGTAAGTGGA
cj0310c-R	AATACAAACCCCAATGGCTGTTA
cj0345-F	TATGGTGTTGTTTTGGGTTCTTC
cj0345-R	CCAGCAATAGGGGCTAAATAAAT
cj0425-F	CTTCAGGCCAACTCATTAGA
cj0425-R	GGGCTTTAAGTCCGGATAATTC
cj0426-F	AACCTTGATTTAGAGGCGATTTC
cj0426-R	GCCTATCATGAGAGATGACAACC
cj0767-F	ATGGTTTCCATATCTTCCCAAAG
cj0767-R	TAATCCGTGGACTTAGAGCAGTG
cj1168c-F	CAAGTATAGCCACCCAAATAGCA
cj1168c-R	TTACCAGGGATTCGTCAGTACAT
cj1170-F	CAAAATCAGTCATATGAGCCACA
cj1170-R	CTATGCTGCAGTAGAAGGAGAGC
cj1173-F	CTGTATGGGAGCTTTTAGGGATT
cj1173-R	TCCAACGATAGAAAGAACAATGC
cj1226-F	GCTTTGCTTCTGTTATCTCATCC
cj1226-R	AGGCTCTTAAATTTTGCAGGAGT
cj1563-F	GCAGTTTTTATACCCAAAGAGCA
cj1563-R	GCGATATAGAATGGGTAAAATGG
Primers for conventional RT-PCR	
cj0423-F	GCGGGTCTATTTTTGTA
cj0425-R	CTTGGCTATTTCCTTGA

*Underlined sequences indicate recognition site for the corresponding restriction endonuclease, which is shown in parenthesis.

### Characterization of *cj1169c-cj1170c* operon

The microarray and qRT-PCR results demonstrated that *cj1169c* and *cj1170c* were up-regulated in both inhibitory and sub-inhibitory treatments with Ery (Tables 3 and 4). *cj1169c* and *cj1170c* encode a putative periplasmic protein and a 50 kDa outer membrane protein precursor, respectively [23]. Recently, *cj1170c* was characterized as an outer-membrane tyrosine kinase, phosphorylating a number of membrane proteins [24]. To identify the role of the two genes in adaptation to Ery treatment, both genes were deleted to produce the mutant strain KOp50Q. The mutation did not affect the transcript abundance of the downstream gene, *cj1168c*, as determined by qRT-PCR (data not shown). The mutant was complemented to produce strain Comp50Q.

The wild-type and mutant strains demonstrated comparable growth rates in MH broth without or with sub-inhibitory (1/2, 1/4, 1/8, and 1/16× MIC) concentrations of Ery (data not shown). Additionally, no significant difference in motility was observed between the mutant and wild-type strains. Furthermore, the MIC test revealed no significant differences between the wild type strain and KOp50Q in susceptibility to a number of antimicrobials including ampicillin, erythromycin, tylosin, ciprofloxacin, tetracycline, phosphonomycin, cetylpyridinium chloride, chloramphenicol, nalidixic acid, novobiocin, ethidium bromide and crystal violet (results not shown). Likewise, as shown by the disk diffusion assay, no significant differences were revealed between the mutant and wild-type strains in sensitivity to oxidative stress agents including H_2_O_2_ and cumene hydroperoxide (data not shown). However, the aerobic stress experiments indicated that the mutant was more susceptible than the wild-type strain to higher levels of oxygen, although they showed comparable growth under microaerobic conditions (Figure 2C). Complementation of the mutant (Comp50Q) partially restored the phenotype to the wild-type level (Figure 2C).

**Figure 4:**
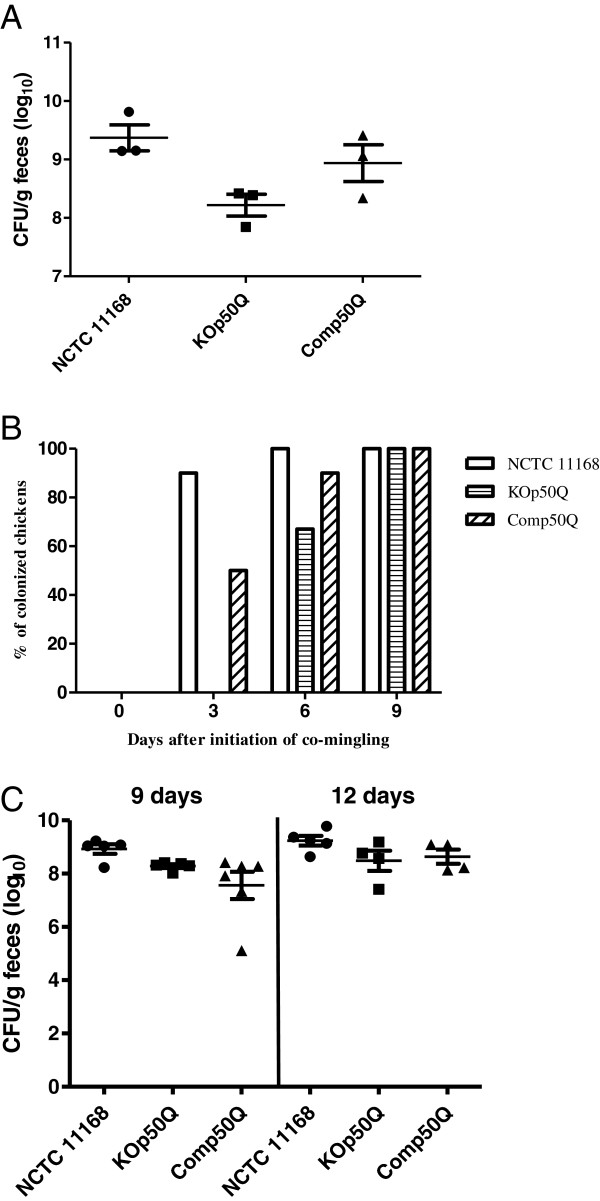
**Effect of mutating the *cj1169c-cj1170c *operon on *Campylobacter *colonization and transmission in birds.** (**A**) Colonization levels in chickens inoculated with wild-type NCTC11168, KOp50Q, and Comp50Q, respectively. The birds were necropsied on 9 and 12 DAI. Each symbol represents a single bird. Horizontal bars indicate the mean and standard error for each group. (**B**) Transmission of *C. jejuni* from seeder birds to naive (non-inoculated) birds. The percentage of naive birds positive for *C. jejuni* in each group was shown. (**C**) Cecal colonization levels of the wild-type, KOp50Q, and Comp50Q in naive birds co-mingled with seeder birds. The birds were sacrificed at 9 and 12 days after initiation of co-mingling. Each symbol represents the colonization level in a single bird. The horizontal bars indicate the mean and standard error for each group.

To determine the role of *cj1169c-cj1170c* in colonization of and horizontal transmission between birds, a co-mingling chicken experiment was performed with wild-type, mutant (KOp50Q) and complement strains (Comp50Q). All 3 seeder birds in each group became *Campylobacter-*positive for the respectively inoculated strain at 3 days after inoculation (DAI) as determined by cloacal swabbing and culturing on selective plates. The three KOp50Q-inoculated seeder birds showed attenuated colonization levels compared with those inoculated with the wild-type strain (*p* = 0.02), while the complement strain resulted in comparable colonization level to that of the wild-type strain (*p* = 0.32) as determined by culturing cecal contents collected at necropsy on 9 or 12 DAI (Figure 4A). The co-mingling experiment showed that 3 days after the initiation of co-mingling, 90% and 50% of the naive (non-inoculated) chickens were colonized by the wild-type and complement strains, respectively, while none of the naive chickens in the KOp50Q group was *Campylobacter* positive on the same day (Figure 4B). This difference was statistically significant (*p* < 0.05). At 6 days after initiation of co-mingling, all of the naive birds in the wild-type group were positive, while 67% of the naive birds were positive in the KOp50Q group and 90% were positive in the complement group. The differences were not statistically significant. At 9 days after initiation of co-mingling, all the naive birds were positive in all three groups as determined by culturing cloacal swabs (Figure 4B). In addition to the cloacal swabs, cecal contents were collected from the naive birds necropsied on 9 and 12 days after initiation of co-mingling to determine colonization levels. At 9 days after initiation of co-mingling, the naive birds colonized by KOp50Q or by Comp50Q had fewer *C. jejuni* than the naive birds colonized by the wild-type strain (Figure 4C) and the difference was statistically significant (*p* < 0.05). At 12 days after initiation of co-mingling, naive birds were colonized by KOp50Q or Comp50Q at similar levels to the wild-type group (*p* > 0.05).

## Discussion

In this study, we determined the transcriptomic changes in *C. jejuni* in response to Ery treatment in an attempt to identify initial molecular mechanisms involved in adaptation to macrolide challenge and resistance development. Wild-type Ery-susceptible *C. jejuni* NCTC 11168 was exposed to different doses of Ery to reveal the adaptive responses to inhibitory and sub-inhibitory antibiotic challenges. In addition to NCTC 11168, its Ery^R^ derivative JL272 strain was also exposed to Ery at a concentration considered inhibitory for the wild-type (4 mg/L). A relatively short treatment period (30 min) was chosen in order to minimize possible collateral effects that might occur from prolonged drug treatment. Transcriptomic analyses revealed a number of genes with altered expression levels in response to Ery treatment, of which the most obvious changes are up-regulation of genes involved in cell motility and down-regulation of genes involved in energy production (Table 1).

Ery and other macrolide antibiotics block the ribosome elongation tunnel to prevent movement and release of the nascent peptide during bacterial protein synthesis. Previous studies have demonstrated that treatment of *E. coli* and *H. influenza* with translation inhibitors (such as puromycin, tetracycline, chloramphenicol, and erythromycin) increased the relative synthesis rate of a number of ribosomal proteins and translation factors as a possible compensating mechanism [12,14]. Consistent with the findings in other bacteria, treatment of *C. jejuni* with an inhibitory dose of Ery increased the transcription of ribosomal proteins, translation initiation factor (IF-1) and transcription elongation factor (*nusA*) (Table 1; Additional file 1). This finding suggests that *C. jejuni* increases transcription of these genes in order to help recover halted peptide elongation and resume translation as its immediate response against the antibiotic exposure. Interestingly, treatment of an Ery^R^ strain (JL272) with a dose of Ery inhibitory for its wild-type ancestor did not trigger noticeable transcriptomic responses. This observation suggests that the 23S RNA mutation in JL272 prevented the interaction of Ery with its target and consequently prohibited the induction of a transcriptomic response in *C. jejuni*.

Of note, several functional gene categories were significantly affected in the wild-type *C. jejuni* by an inhibitory dose of Ery (Table 1), suggesting that *C. jejuni* alters multiple pathways to cope with Ery stress. Most of the differentially expressed genes in the COG category “energy production and conversion” were down-regulated (Table 1), suggesting that reduced energy metabolism occurred as an adaptive response to inhibitory treatment with Ery. This result is consistent with findings in other bacteria such as *Staphlococcus aureus*, *E. coli*, and *Y. pestis,* which demonstrated significant down-regulation of “energy metabolism” genes under treatment with different classes of antibiotics [15-17]. Taken together, these observations suggest that reduced energy metabolism may be a general transcriptional response to antibiotic-induced stress in both Gram-positive and Gram-negative bacteria. Other COG categories with a noticeably high proportion of down-regulated genes (as compared with the proportion of up-regulated genes in the same categories) included “cell wall/membrane biogenesis”, “carbohydrate transport and metabolism”, and “nucleotide transport and metabolism” (Table 1 and Additional file 1). These changes suggest that *C. jejuni* decreased the general metabolic rates to prolong the survival time under Ery challenge.

Genes involved in “transcription” and “translation” was noticeably up-regulated. In the COG category of “transcription”, up-regulated genes included flagellar biosynthesis sigma factor (*fliA*), putative transcriptional regulator (*cj1563c*), transcription elongation factor NusA (*nusA*) and heat-inducible transcription repressor (*hrcA*) (Additional file 1). Among the up-regulated genes in the “translation” category included 50S ribosomal protein L1 (*rplA*), L20 (*rplT*), 30S ribosomal protein S2 (*rpsB*), and translation initiation factor IF-1 (*infA*) (Additional file 1). Since Ery targets 50S ribosomal proteins and block the ribosome elongation tunnel, this finding suggests that *C. jejuni* increases transcription of these genes in order to help recover the halted peptide elongation and resume translation as its immediate response against the antibiotic exposure. In the “Defense mechanism” category, two genes were up-regulated after inhibitory treatment, which encode putative MATE family transport protein (*cj0619*) and ABC-type transmembrane transport protein (*cj0607*). The role of these genes in the adaptation to Ery treatment remains undetermined.

The “cell motility” category comprised the largest proportion of up-regulated genes in response to an inhibitory dose of Ery in wild-type *C. jejuni* (Table 1), suggesting that enhanced motility might be *Campylobacter*’s initial escape response to this noxious stress. *cj0061c,* which encodes the σ^28^ transcription factor *fliA* and is essential for normal flagellar biosynthesis [25], is up-regulated in NCTC 11168 when treated with inhibitory and sub-inhibitory doses of Ery (Table 3). This gene induction was independently confirmed by qRT-PCR (Table 4). Previous research indicated that σ^28^ regulates the major flagellin gene (*flaA*) and other late genes of the flagellar regulon as well as some non-flagellar genes in *C. jejuni*[26]. Also, it has been demonstrated that the *flaA* promoter can be activated by the intestinal environment and *C. jejuni* chemotactic effectors, such as bovine bile, deoxycholate, L-fucose, osmolarity, aspartate, glutamate, organic acids citrate, fumarate, α-ketoglutarate and succinate [27]. The microarray and qRT-PCR results presented here revealed that Ery induced expression of this regulatory gene (*fliA*), which might explain why multiple motility genes were up-regulated in *C. jejuni* under Ery treatment.

Compared with the inhibitory-dose Ery treatment, sub-inhibitory dose Ery triggered a much smaller response in the overall transcription in *C. jejuni* (Table 2 and Additional file 1). There were no or limited changes in most COG categories, except for “poorly characterized” and “amino acid transport and metabolism”. For example, no differentially expressed genes were found in the “energy production and conversion” category under sub-inhibitory Ery treatment (Table 2), while a large portion of genes in this category were down-regulated under the treatment of an inhibitory does of Ery (Table 1). In the “cell motility” category, only two genes were up-regulated under the sub-inhibitory Ery treatment, but a number of genes in this category were up-regulated in response to an inhibitory dose of Ery (Table 1). Additionally, no genes in the “translation” category were altered in expression under the sub-inhibitory dose, but multiple genes in this category were up-regulated when treated with an inhibitory dose. These differences suggest that the sub-inhibitory dose of Ery did not significantly affect the fundamental metabolism of *C. jejuni*. Despite these major differences, there were 14 genes that showed consistent trends of differential expression under both inhibitory and sub-inhibitory treatments (Table 3). Among the 14 genes include a two-component sensor kinase (*cj1226c*), *omp50* (*cj1170c*), and *fliA* (*cj0061c*). Interestingly, several COG categories did not show any appreciable gene expression changes regardless of the doses of Ery exposure. These categories include cell “cycle control, mitosis and meiosis”, “intracellular trafficking and secretion” as well as those involved in transport and metabolism of lipids and nucleic acids (Tables 1 and 2). Together, these findings suggest that Ery exposure invokes transcriptional responses that are more prominent in certain metabolic pathways and are influenced by the doses of the antibiotic.

Several differentially expressed genes were selected for detailed studies by generating insertional mutants in the study. The selection was based on their predicted or known functions (for the PMSR genes and the *cj1169c-cj1170c* operon) or the magnitude of differential expression (for the *cj0423-cj0425* operon). Interestingly, mutation of these selected genes did not affect the susceptibility of *C. jejuni* to Ery, although their expression was up-regulated in the presence of this antibiotic. This finding suggests that these genes are involved in the response to Ery treatment, but may not contribute directly to macrolide resistance. Alternatively, these genes may contribute to Ery resistance when they are over expressed. This possibility is not examined in this study and remains to be evaluated. Additionally, functional redundancy of genes may compensate for the inactivation of the selected genes, preventing an obvious change in the susceptibility to Ery.

PSMR transporters in other bacteria have been demonstrated to confer resistance to numerous toxic compounds including quaternary ammonium compounds, toxic lipophilic compounds, potentially toxic metabolites and polyamine compounds [21,28,29]. Not all PSMR proteins are associated with an antibiotic resistance phenotype [34], highlighting the diversity in substrate recognition by PSMR transporters. In *C. jejuni*, the substrates recognized and exported by Cj0309c-Cj0310c and Cj1173-Cj1174 remain unknown. However, their mutants showed reduced survival compared to the wild-type strain at 18.5% O_2_ (Figure 2A), suggesting that the PSMR proteins may contribute to *Campylobacter* survival under high-level oxygen tension such as the conditions encountered outside of the host during transmission. However, the chicken experiments demonstrated that the mutant strains were comparable to the wild-type strain in the ability to colonize and spread among birds (Figure 3), suggesting that the *Campylobacter* PSMR transporters are not essential for *in vivo* colonization and transmission. One potential caveat of the chicken experiment is the short-term nature of the study and the continuous shedding of fresh *Campylobacter* (from the seeder birds) that were available for the naïve birds, which may not allow evaluation of the role of the PSMR genes in long-term survival and transmission. This possibility requires further examination in future studies.

*cj0425* was identified as up-regulated (>100 fold) by microarray when *C. jejuni* was treated with an inhibitory dose of Ery (Additional file 1), and qRT-PCR confirmed this change (Table 4). In this study, we provided empirical evidence that *cj0423-cj0425* are co-transcribed from the same operon (data not shown). Little is known about the function of this operon. Previously, it was demonstrated that *cj0425* (encoding a putative periplasmic protein) was down-regulated under low oxygen conditions and is considered to be involved in oxidative-tolerance phenotype of *C. jejuni*[30,31]. However, it is shown in this study that *C. jejuni* wild-type NCTC 11168 and its Δ*cj0425* isogenic mutant strain (KO423Q) had comparable level of resistance to the oxidative stress generating compounds tested in this study (result not shown), suggesting that it is not directly involved in oxidative stress resistance.

Omp50 (*cj1170c*) of *C. jejuni* was previously characterized to belong to the monomeric group of porins which is typical of the OmpA-like family [23]. Omp50 was also found to be species-specific and present only in *C. jejuni* and *C. lari*, but not in *C. coli*[32]. Previous studies showed that the temperature regulated Omp50 maybe an alternative porin to the major outer membrane protein (MOMP), contributing to decreased membrane permeability while still allowing nutrient uptake [33,34]. However, a recent study identified Omp50 as an outer-membrane phosphotyrosine kinase that modulates phosphorylation of multiple outer membrane proteins and carbohydrate biosynthesis in *C. jejuni*[24]. Specifically, Omp50 positively regulates UDP-GlcNAc/Glc 4-epimerase, which is required for N-glycosylation, capsule production and virulence. In this study, it was found that expression of Omp50 and the downstream gene *cj1169c* was up-regulated in response to both high and low doses of Ery treatment (Tables 3 and 4). This up-regulation could be an adaptive response as increasing expression of surface polysaccharides is expected to reduce cell permeability to Ery, which is a hydrophobic antibiotic. Additionally, it was shown in this study that the Omp50 mutant (KOp50Q) was less tolerant than the wild-type to high levels of oxygen (Figure 2C), showed reduced colonization in chickens, and delayed transmission between seeder birds and non-inoculated birds (Figure 4). These phenotypic changes could be explained by the role of Omp50 in protecting *Campylobacter* against reactive oxygen species produced by host intestinal epithelium [24] and against high oxygen tension encountered during fecal-oral transmission. Together, these findings suggest that the *cj1169c-cj1170c* operon contributes to *Campylobacter* adaptation *in vitro* and in animal hosts.

## Conclusions

In summary, the findings from this study indicate that Ery treatment of *C. jejuni* elicits a transcriptomic response that affects a wide range of functional categories. The most notable changes are up-regulation of motility genes and down-regulation of genes involved in energy production and conversion. The transcriptomic response is influenced by the doses of Ery and is prevented by the resistance-conferring mutation in the 23S RNA. Inactivation of several selected genes did not affect the susceptibility of *C. jejuni* to Ery, but some of the mutant strains showed reduced tolerance to oxygen *in vitro* and decreased colonization in chickens. Together, these results suggest the adaptive responses may contribute to the survival of *C. jejuni* under antibiotic stress and facilitate the development of Ery-tolerant/resistant variants.

## Methods

### Strains, media, and growth conditions

Bacterial strains and plasmids used in this study are listed in Table 5. *Campylobacter* strains were routinely cultured from frozen stocks (−80°C) on Mueller-Hinton (MH) agar or broth at 42°C under microaerobic conditions (85% N_2_, 10% CO_2_ and 5% O_2_). For oxygen-stress experiments, the strains were grown on MH agar under an increased oxygen containing atmosphere (76.5% N_2_, 5% CO_2_, and 18.5% O_2_) at 37°C. *E. coli* was grown in Luria-Bertani (LB) broth or agar at 37°C. The media was supplemented with chloramphenicol (4 mg/L; ACROS), kanamycin (30 mg/L; Sigma), or tetracycline (5 mg/L; Sigma) when needed.

### Growth rate and antibiotic susceptibility test

To assess *in vitro* growth, *C. jejuni* strains were inoculated into MH broth to a density of 10^7^ CFU mL^-1^ and incubated with shaking (160 rpm) at 42°C under microaerobic conditions. Optical density at 600 nm (OD_600_) was monitored by a spectrophotometer (Bio-Rad smartspec™3000, Hercules, CA) at various time points (2 h, 4 h, 6 h, and 8 h post inoculation).

The minimum inhibitory concentrations (MIC) of Ery and other antimicrobials for NCTC 11168 and its mutant strains were determined by a microtiter broth dilution method as described previously [35]. The antibiotics and compounds were purchased from Sigma (ampicillin, Ery, streptomycin, novobiocin, nalidixic acid, tetracycline, phosphonomycin, cetylpyridinium chloride), Fisher Scientific (crystal violet, erythromycin), ACROS (chloramphenicol), IBI Scientific (ethidium bromide (EB)), Fluka (ciprofloxacin), Ambion (SDS), and Alfa Aesar (spermidine). Results were recorded after 24 h incubation under microaerobic conditions at 42°C. Tests for each compound were repeated three times.

### DNA microarray experiments

Wild-type *C. jejuni* NCTC 11168 (Ery MIC: 0.25 mg/L) and its erythromycin-resistant (Ery^R^) derivative strain JL272 (Ery MIC: 1024 mg/L) [36] were grown separately for 5 hours in MH to OD_600_ of approximately 0.2 with shaking (160 rpm) at 42°C under microaerobic condition. Fifteen mL aliquots of NTCT 11168 culture (in triplicates) were treated with either sham (ethanol solvent for Ery), an inhibitory dose of Ery (4 mg/L; 16× MIC), or a sub-inhibitory dose of Ery (0.125 mg/L; 0.5× MIC). All cultures including the sham control were thoroughly mixed and statically incubated under microaerobic conditions for 30 minutes at 42°C. Strain JL272 was treated with 4 mg/L Ery (16× MIC of the wild-type strain) or the sham under the same condition as with NCTC 11168. After 30 minutes treatment, the cultures were immediately mixed with RNAprotect™ (Qiagen, Valencia, CA) to stabilize the total bacterial RNA. Total RNA was extracted using the RNeasy Mini kit (Qiagen) according to the manufacturer’s protocol and treated with TURBO DNase (Invitrogen, Carlsbad, CA). RNA quantity was determined by OD_260_ reading using a NanoDrop spectrometer (Thermo Scientific, Wilmington, DE), and the purity was assessed by denaturing agarose gel electrophoresis. RNA samples confirmed free of DNA contamination by PCR of 16S rRNA gene, were stored at −80°C until use. Three independent RNA isolations (biological replicates) were performed for microarray experiments.

*C. jejuni* microarray slides (version 3 for NCTC 11168 inhibitory treatment, version 4 for NCTC 11168 sub-inhibitory treatment, and version 1 for JL272 Ery treament) were designed and provided by the Pathogen Functional Genomics Resource Center (PFGRC) at the J. Craig Venter Institute (JCVI, Rockville, MD). cDNA synthesis, labeling of cDNA and hybridization of labeled cDNA to the microarray slides were performed according to the JCVI’s protocol (http://pfgrc.jcvi.org/index.php/microarray/protocols.html). For each pair of treated and untreated samples, hybridizations were performed with RNA samples prepared from three independent experiments, with the cDNA alternately labeled with Cy3 and Cy5 for the pair in each slide.

Slides were dried using a microarray high speed centrifuge (Arrayit, Sunnyvale, CA) and immediately scanned at a wavelength of 550 nm for Cy3 and 650 nm for Cy5 using a General Scanning ScanArray 5000 (PerkinElmer, Boston, MA) at 10 μm resolution. Slide information and annotation files were obtained from the JCVI website (http://pfgrc.jcvi.org/index.php/microarray/available_microarrays/.html). The fluorescence intensities were collected and converted to digital signal by ImaGene software (BioDiscovery, EI Segundo, CA). The fluorescence intensity values were logarithm-transformed, median background corrected, and LOWESS normalized. The normalized gene expression data were analyzed using moderated-*t* test implemented in the R package, LIMMA [15]. In this study, a *p-*value < 0.01, relative fold-change ≥ 2 were chosen as the cutoff for identification of genes with a significant differential expression between the treatment and control samples. The microarray data have been deposited in the NCBI Gene Expression Ommibus (http://www.ncbi.nlm.nih.gov/gds/) and the accession number is GSE43026.

### Quantitative real-time RT-PCR

A quantitative real-time RT-PCR (qRT-PCR) was used to confirm the expression levels of representative genes that were identified as differentially expressed by the microarray. Briefly, reactions were performed using the iQTM SYBRR Green Super Mix (Bio-Rad, Hercules, CA) and MyiQTM instrument (Bio-Rad). Primers were designed by Primer 3 software (http://frodo.wi.mit.edu/) and are listed in Table 6. The 16S rRNA transcript was used to normalize target gene expression. Amplification efficiency and relative transcript abundance (R) were calculated as previously described [37]. R values were log_2_ transformed to meet assumptions of normality and variance; statistical significance was determined by the two tailed Student’s *t*-test under the null hypothesis of R = 0.

### Construction and complementation of insertional mutants

Isogenic *C. jejuni* NCTC 11168 mutant strains with a disrupted copy of *cj0309c-cj0310c*, *cj0423-cj0425*, *cj1169c-cj1170c,* or *cj1173-cj1174* genes were constructed by insertional mutagenesis with antibiotic resistance cassettes. The strategies are shown in Figure 1. Primers used in the construction and complementation of mutants are listed in Table 6. The chloramphenicol (*cat*) and kanamycin (*aphA-3*) resistance cassettes were PCR amplified using Ex-Taq (Takara Bio Inc.) from plasmids pUOA18 and pMW10 with *cat* and *aphA3* primers, respectively, as described in a previous study [38]. PCR products were digested with the appropriate restriction enzymes (Table 6, Figure 1). The PCR products and a resistance cassette were ligated by T4 DNA ligase (Promega, Madison, WI), cloned into suicide vector pUC19 (Invitrogen, Carlsbad, CA), and transformed into competent *E. coli* DH5α (Invitrogen). Recombinant clones with the intended mutation were confirmed by PCR. Plasmids were extracted from DH5α and used to transform wild-type NCTC 11168 by the standard biphasic method for natural transformation [39]. Transformants were colony purified on MH plates with supplemented antibiotics. Single colonies were selected and confirmed by PCR. Mutations were complemented by inserting the entire set of the wild-type copy of genes between the structural genes of the ribosomal gene cluster in the corresponding mutant strains as described previously [37,40]. PCR amplification and sequencing were performed on positive clones to confirm no mutations occurred in the cloned sequences. All strains were stored at −80°C for later use.

### Oxidative stress tests

To determine if the mutated genes affected the susceptibility of *C. jejuni* to oxidative stress, wild-type NCTC 11168 and mutant strains (KO39Q、KO73Q、KO425Q、KOp50Q and DKO01Q) were compared using two oxidative stress tests. In the first test, inhibition of hydrogen peroxide (H_2_O_2_), cumene hydroperoxide and menadione on bacterial growth at 24 and 48 h on MH plate incubated microaerobically at 42°C were measured by a disk diffusion method as described previously [41], with the following modification: The concentrations of H_2_O_2,_ cumene hydroperoxide and menadione used were 1.5%, 2% and 45 mM, respectively. In the second test, oxygen tolerance of wild-type and mutant strains was determined by measuring the viability/growth after incubation at different oxygen levels (5% O_2_ or 18.5% O_2_) as described previously [42] with modifications. Briefly, serial dilutions of overnight cultures were spotted (5 μl) onto MH agar plates and incubated at 37°C in incubators containing either 5% O_2,_ 10% CO_2_, 85% N_2_ or 18.5% O_2,_ 5% CO_2_, 76.5% N_2_ (Forma Scientific, model 3130). Growth was examined after 48 h of incubation. Experiments were repeated three times independently.

### Colonization and transmission experiments in chickens

To investigate if *cj0309c-cj0310c* and *cj1173-cj1174*, which encode putative multidrug efflux systems, affect *Campylobacter* adaptation in chickens, 3-day-old commercial broiler chickens (Ross & Ross) were randomly assigned to 4 groups (15 bird/group) and inoculated with NCTC 11168 (group 1), KO39Q (Δ*cj0309c-cj0310*c, group 2), KO73Q (Δ*cj1173-cj1174*, group 3), and DKO01Q (Δ*cj0309c-cj0310* and Δ*cj1173-cj1174*, group 4), respectively. Each bird received approximately 1x10^7^ CFU of respective strain via oral gavage. The birds were free of *Campylobacter* colonization as determined by culturing of cloacal swabs prior to inoculation. Cecal contents were collected from each bird at necropsy on 5, 10, and 15 DAI. The total number of *Campylobacter* in each sample was determined by serial dilution and viable counts on agar plates containing *Campylobacter*-specific growth and selective supplements (Oxoid, United Kingdom). The samples from groups 2, 3, and 4 were also plated on *Campylobacter*-selective agar plates containing kanamycin or/and chloramphenicol as described earlier to confirm the mutations. *Campylobacter* counts were determined after 48 h incubation microaerobically at 42°C, and expressed as CFU/g feces for each bird at each sampling point.

In addition to the colonization experiment described above, co-mingling experiments were carried out to determine the transmissibility of mutant strains from *Campylobacter-*inoculated seeder birds to naive (non-inoculated) birds. The strains used in this study included the wild type strain NCTC 11168 (group 1), DKO01Q (Δ*cj0309c-cj0310c* and Δ*cj1173-cj1174*,group 2), KOp50Q (Δ*cj1169c-cj1170c*,group 3), and Comp50Q (complemented KOp50Q strain, group 4). One-day-old commercial broiler chickens (Ross & Ross) were randomly assigned to four groups (n = 12 for groups inoculated with KOp50Q or DKO01Q; n = 13 for the groups with NCTC 11168 or Comp50Q), which were segregated by cardboard pens in separate rooms. Three birds (marked with a permanent marker) in each group were randomly chosen and inoculated with a dose of 10^7^ CFU of respective strain via oral gavage at day 3 of age. After inoculation, the inoculated birds were immediately returned to the respective groups and allowed to comingle with non-inoculated birds. Cloacal swabs were collected from each bird at 3, 6, and 9 DAI for determining the positivity (with *Campylobacter*) of the birds. Additionally, the birds were necropsied at 9 and 12 DAI (n = 6 or 7 for each time point) and the cecal contents were collected for measuring the level of colonization. It should be pointed out that in terms of time frame the DAI were the same as days after initiation of comingling as the co-mingling occurred immediately after inoculation of the seeder birds. Cloacal swabs were streaked on the selective agar media to determine *Campylobacter* presence/absence. Cecal contents were serially diluted and tested to quantify *Campylobacter* colonies as described above.

The detection limit of the culture method used for the chicken experiments was 100 CFU/g of feces. Cecal contents contained less than 100 CFU/g *Campylobacter* colonies were considered negative and assigned a value of 0 for the purpose of statistical analysis. Significant differences (*p* < 0.05) in the colonization levels between groups at each sampling time point were determined using Student’s *t* test, Welch's *t* test to allow for non-constant variation across treatment groups, and the Wilcoxon rank-sum test to allow for non-normality [11].

All animals used in this study were handled in strict accordance with the recommendations in the Guide for the Care and Use of Laboratory Animals of the National Institutes of Health. The animal use protocol was approved by the Institutional Animal Care and Use Committee of Iowa State University (A3236-01). All efforts were made to minimize suffering of animals.

## Competing interests

The authors declare that they have no competing interests.

## Authors’ contributions

QX carried out the experiments, conducted data analysis, and drafted the manuscript. WM participated in experimental design, chicken experiment, and statistical analysis, and helped to draft the manuscript. ZS constructed the KO39Q mutant and participated in microarray data analysis and chicken experiments. OS participated in chicken experiments and helped to draft the manuscript. HW participated in the design of the study and helped to draft the manuscript. ZW participated in microarray experiments analysis and helped to draft the manuscript. PL contributed to the design of the microarray experiment and data analysis. QZ conceived the study, participated in experimental design and data analysis, and revised the manuscript. All authors have read and approved the final manuscript.

## Supplementary Material

Additional file 1: Table S1. Up-regulated genes in *C. jejuni* NCTC 11168 in response to treatment with an inhibitory dose of Ery. **Table S2:** Down-regulated genes in *C. jejuni* NCTC 11168 in response to treatment with an inhibitory dose of Ery. **Table S3:** Up-regulated genes in *C. jejuni* NCTC 11168 in response to treatment with a sub-inhibitory dose of Ery. **Table S4:** Down-regulated genes in *C. jejuni* NCTC 11168 in response to treatment with a sub-inhibitory dose of Ery. Click here for file
